# GABA Coordinates with Insulin in Regulating Secretory Function in Pancreatic INS-1 β-Cells

**DOI:** 10.1371/journal.pone.0026225

**Published:** 2011-10-21

**Authors:** Paul Bansal, Shuanglian Wang, Shenghao Liu, Yun-Yan Xiang, Wei-Yang Lu, Qinghua Wang

**Affiliations:** 1 Departments of Physiology and Medicine, Faculty of Medicine, University of Toronto, Toronto, Ontario, Canada; 2 Division of Endocrinology and Metabolism, The Keenan Research Centre in the Li Ka-Shing Knowledge Institute, St. Michael's Hospital, Toronto, Ontario, Canada; 3 Department of Physiology and Pharmacology, University of Western Ontario, London, Ontario, Canada; 4 Robarts Research Institute, University of Western Ontario, London, Ontario, Canada; University of Bremen, Germany

## Abstract

Pancreatic islet β-cells produce large amounts of γ-aminobutyric acid (GABA), which is co-released with insulin. GABA inhibits glucagon secretion by hyperpolarizing α-cells via type-A GABA receptors (GABA_A_Rs). We and others recently reported that islet β-cells also express GABA_A_Rs and that activation of GABA_A_Rs increases insulin release. Here we investigate the effects of insulin on the GABA-GABA_A_R system in the pancreatic INS-1 cells using perforated-patch recording. The results showed that GABA produces a rapid inward current and depolarizes INS-1 cells. However, pre-treatment of the cell with regular insulin (1 µM) suppressed the GABA-induced current (I_GABA_) by 43%. Zinc-free insulin also suppressed I_GABA_ to the same extent of inhibition by regular insulin. The inhibition of I_GABA_ occurs within 30 seconds after application of insulin. The insulin-induced inhibition of I_GABA_ persisted in the presence of PI3-kinase inhibitor, but was abolished upon inhibition of ERK, indicating that insulin suppresses GABA_A_Rs through a mechanism that involves ERK activation. Radioimmunoassay revealed that the secretion of C-peptide was enhanced by GABA, which was blocked by pre-incubating the cells with picrotoxin (50 µM, p<0.01) and insulin (1 µM, p<0.01), respectively. Together, these data suggest that autocrine GABA, via activation of GABA_A_Rs, depolarizes the pancreatic β-cells and enhances insulin secretion. On the other hand, insulin down-regulates GABA-GABA_A_R signaling presenting a feedback mechanism for fine-tuning β-cell secretion.

## Introduction

Gamma-aminobutyric acid (GABA) is a major neurotransmitter in the central nervous system (CNS), where GABA produces fast inhibition in mature neurons primarily by activation of A-type GABA receptor (GABA_A_R), a hetero-pentameric Cl^-^ channel [1]. A large amount of GABA is also produced in the pancreatic islet [2], where it exists at the highest concentration outside of the CNS [3]. Pancreatic GABA is primarily produced by the β-cell [4], in which GABA is stored in synaptic-like microvesicles that are distinct from insulin-containing large-dense core vesicles (LDCVs) [5]. However, recent evidence indicates that GABA is co-localized with insulin in LDCVs in human islets and that the release of GABA from the β-cells is glucose-dependent [6]. The release of GABA from β-cells is “tonic” [7], [8], yet the amount of released GABA is regulated by the metabolic state of β-cells [9].

In the pancreatic islet, GABA released from β-cells plays a critical role in the regulation of glucagon secretion from α-cells. Specifically, GABA activates GABA_A_Rs in α-cells, sequentially leading to an influx of Cl^-^ and membrane hyperpolarization, and hence an inhibition of glucagon secretion. The GABA_A_R-mediated hyperpolarization of α-cells represents a physiological mechanism for glucose-induced suppression of glucagon release because blockade of GABA_A_R diminishes the inhibitory effect of high glucose on glucagon secretion in isolated rat [10] or mouse [11] islets. In relation to this notion, we have recently demonstrated that insulin suppresses glucagon secretion by enhancing intra-islet GABA-GABA_A_R signaling through translocation of GABA_A_R from an intracellular pool to the cell surface of α-cells [12].

Studies, including ours, have demonstrated that GABA_A_Rs are also expressed in the primary islet β-cells [12], [13] and insulin-secreting clonal β-cell lines [14], [15]. Unlike in mature neurons and α-cells, stimulation of GABA_A_Rs in β-cells induces membrane depolarization, enhancing insulin secretion in the presence of physiological concentrations of glucose [6], [15]. Consistent with the notion that the autocrine insulin is essential for β-cell function [16], [17], we recently demonstrated that GABA, in cooperation with insulin, enhances the proliferation and survival of the β-cells through activation of the PI3-K/Akt pathway. Remarkably, GABA promotes β-cell regeneration and reverses diabetes in mouse models [18]. In the present study, we found that insulin negatively regulates GABA_A_R function and inhibits GABA-induced β-cell secretion. Our results demonstrated a feedback mechanism that fine-tunes β-cell secretion.

## Materials and Methods

### Cell culture

Rat insulinoma INS-1 cells (passage 50–65) were maintained in RPMI 1640 medium (Invitrogen, Burlington, ON, Canada) containing fetal bovine serum (10% v/v), 100 Units/ml penicillin G sodium, 100 µg/ml streptomycin sulphate, 55 mg/500 ml sodium pyruvate, 1.14 g/500 ml HEPES, and 1.7 µl/500 ml β-mercaptoethanol at 37°C in an atmosphere of humidified air (95%) and CO_2_ (5%). Four hours before being used for patch-clamp recordings, INS-1 cells were glucose-starved in serum-free RPMI 1640 medium that contained 1.4 mM glucose.

### Electrophysiology

For electrophysiological recordings, cells were bathed in the standard extracellular solution (ECS) containing (in mmol/l) 145 NaCl, 1.3 CaCl_2_, 5.4 KCl, 25 HEPES and 1.4 glucose (pH 7.4, 320–340 mOsm), and the ECS was maintained at 30°C. Patch-clamp recordings were performed using an Axopatch-1D amplifier (Axon Instruments, Foster City, CA, USA). Electrodes (1.8–2.3 MΩ) were constructed from thin-walled glass (1.5 mm diameter, World Precision Instruments, Sarasota FL, USA) using a two-stage puller (PP-830; Narshige, East Meadow NY, USA). The standard intracellular solution (ICS) consisted of (in mmol/l) 150 KCl, 10 KOH, 10 HEPES, 2 MgCl_2_ and 1 CaCl_2_ (ATP-free). The pore-forming agent gramicidin (60 mg/ml, Sigma-Aldrich Corp., Buchs, Switzerland) [19] was included in the ICS to perforate the membrane patch of the recorded cell. Under voltage-clamp mode, the membrane perforation was observed as a constant decrease in serial resistance after the electrode seal. In most of the recordings, the resistance declined to a value ranging from 28 to 301MΩ within 5–15 min after the seal, and then stabilized for 45–80 min. All perforated patch recordings began when the serial resistance had attained values below 30 MΩ. To monitor a possible formation of whole-cell configuration, a testing voltage-ramp (a gradual voltage-change from -100 to 100 mV in 1.5 s) was applied to the cell at the start of the recording. With this testing protocol, a sigmoid-shaped current-voltage (I–V) curve was seen under stable perforated patch recordings, whereas a large linear I–V relationship appeared after whole-cell configuration due to the activation of K_ATP_ channels by dilution of the cytosolic ATP. The endogenous membrane potential of INS-1 cells was about −60 mV [20]. Thus, INS-1 cells were voltage-clamped at −60 mV while under constant perfusion by fresh ECS. Patch-clamp recording was performed under voltage- or current-clamp mode. Via a computer-controlled multi-barreled perfusion system (SF-77Bl Warner Instruments, Hamden, CT, USA), 30 µM GABA [the EC50 of GABA in INS-1 cell is 22.3 µM [15]] was briefly (6 s) applied to the patched cells in two-minute intervals. After four stable recordings of GABA-induced current (I_GABA_), insulin was added to the standard ECS. All electrical signals were digitized, filtered (30 kHz), and acquired on-line using the program Clampex and analysed off-line using the program Clampfit 9 (Axon Instruments).

### Measurement of intracellular calcium levels

Cells cultured in 96-well plates were loaded with 5 µM Fluo-3 AM (Molecular Probes, Eugene, OR, USA) for 2 hours in Locke's Buffer. Cells were then treated with GABA (30 µM), or 5 mM KCl as positive control. Changes in relative fluorescence units (RFU) were monitored with the Fluoroskan Ascent FL fluorescent plate reader equipped with a micro-injection syringe pump (Labsystems, Helsinki, Finland), based on the method provided by the manufacturer (Molecular Probes and Labsystems Fluo-3 AM Application Note).

### Plasmids transfection

Dominant negative Akt (DN-Akt) vector was constructed as described previously [12], Green fluorescent protein (GFP)-expressing vector (Invitrogen) was used as an indicator of transfection. INS-1 cells transfected with or without relevant plasmids using Lipofectamine™ 2000 (Invitrogen, 24 hrs) according to manufacture's instruction.

### Western blot analysis

Cells were serum-starved (16 hrs) and treated with or without insulin (100 nM) for 10 min, or in the presence of PI3-K inhibitor wortmannin (100 nM) or MEK/ERK inhibitor PD98059 (20 µM). Cells were lysed in RIPA lysis buffer containing the protease inhibitors phenylmethylsulphonylfluoride (PMSF) (1 mol/l) and EDTA (1 mol/l), Na3VO4 (1 mol/l), and NaF (1 mol/l). Protein of 25 µg was resolved by SDS-PAGE, transferred to nitrocellulose membranes and probed by anti-Akt and anti-phospho-Akt, or anti-ERK1/2 and anti-phospho-ERK1/2 (1∶1,000, Cell Signaling) as described previously [12] **.**

### Insulin secretion

INS-1 cells were plated in 24-well plates with a density of 2.5×10^5^ cells/well in RPMI 1640 medium containing 10% FBS. The following day, the medium was replaced with fresh KRB buffer (containing, in mmol/l, 115 NaCl, 5 KCl, 24 NaHCO_3_, 2.5 CaCl_2_, 1 MgCl_2_, 10 HEPES, 1.4 glucose, and 0.1% BSA) for 60 min. The cells were then treated with 1.4 or 11.1 mM glucose in KRB buffer for 2 h, in the presence or absence of GABA (30 µM). To determine the effects of insulin and/or GABA on the INS-1 cell secretion, in some parallel assays, cells were pre-treated with insulin (Novolin Toronto, Novo Nordisk, 1 µM) or picrotoxin (50 µM) for 15 min prior to GABA treatment during the secretion assay. The insulin levels in conditioned KRB buffer were evaluated by measuring C-peptide using a rat C-peptide RIA kit (Linco Research, St. Louis, MO, USA), according to the manufacturer's instructions.

### Confocal Imaging

INS-1 cells were grown on poly-D-lysine (Sigma)-coated 8-well chamber slides (BD Falcon). Serum-starved cells were pre-treated with or without wortmannin (100 nM, 15 min) and incubated with or without insulin (1 µM) for 15 min). Cells were fixed with 4% paraformaldehyde and blocked with 2% BSA in PBS containing 0.1% Triton X-100 at room temperature for 1 hour. The cells were then incubated with monoclonal mouse anti-GABA_A_R β_2/3_ subunit (UBI 1∶100) and Cy3-conjugated anti-mouse IgG (Jackson Labs, 1∶500), consecutively. The images were captured using a Leica TCS 4D laser confocal fluorescence microscope.

### Statistical Analysis

I_GABA_ is expressed as mean current normalized to the current amplitude obtained immediately preceding insulin treatment (in all cases the fourth sweep in the series of same-cell current recordings) ± SEM. All data were presented as mean ± SEM. Statistical analysis was performed using unpaired or paired Student's t-test where appropriate. A *p*-value<0.05 was considered as significant. Statistical analyses were performed using SigmaPlot 2002 from SPSS Inc. (Chicago, IL, USA) and Microsoft Excel from Microsoft Corp. (Redmond, WA, USA).

## Results

### GABA induces membrane depolarization and increases intracellular Ca^2+^ levels in INS-1 cells

**Figure 1:**
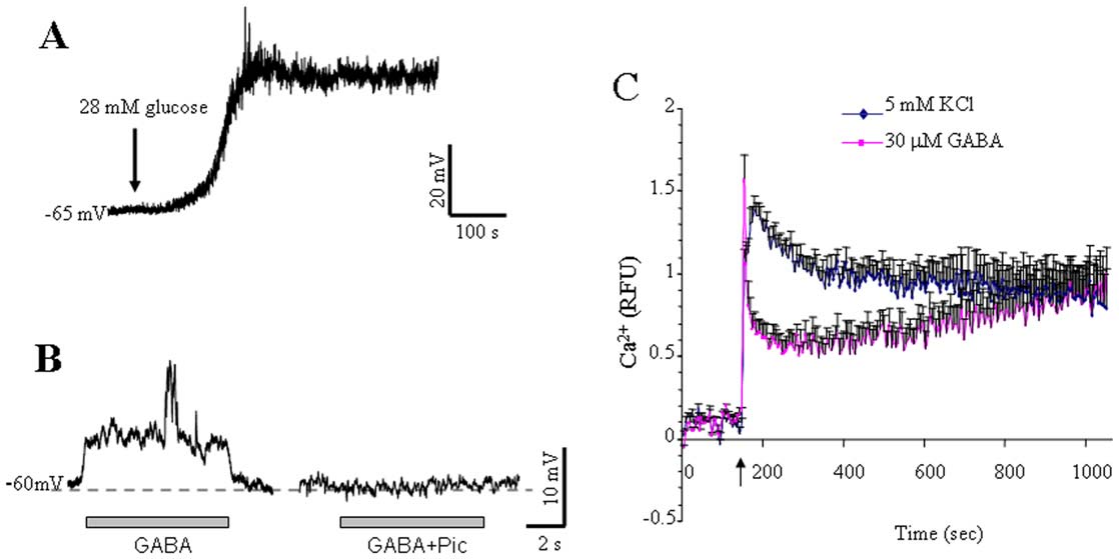
GABA depolarizes membrane potential and increases intracellular Ca^2+^ in INS-1 cells. (A) Perfusion of ECS containing 28 mM glucose induces a gradual and sustained depolarization of the membrane potential (V_m_) (n = 5). (B) GABA induces a rapid and GABA_A_R inhibition-sensitive depolarization of V_m_ under the current-clamp conditions at 1.4 mM glucose (n = 5). (C) Cells cultured in 96-well plates pre-loaded with Fluo-3 AM were treated with GABA (30 µM), or 5 mM KCl as positive control. Changes in relative fluorescence units (RFU) were monitored with a fluorescent plate reader. Data are Mean±SE, n = 6.

As previously demonstrated [20], under current-clamp conditions INS-1 cells displayed a quiescent membrane potential around −60 mV when ECS contained 1.4 mM glucose (**Figure 1A**). Perfusion of the cell with ECS containing 28 mM glucose caused a gradual and sustained depolarization. In some cases, bursts of action potentials were superimposed on the glucose-induced depolarization (**Figure 1A**). Under the same recording conditions, perfusion of GABA induced a fast membrane depolarization in the INS-1 cell (**Figure 1B**). The GABA-induced depolarization was completely blocked by GABA_A_R antagonist picrotoxin (**Figure 1B**) or largely attenuated by bicuculline (not shown), consistent with our previous findings, and those of others in the same cell line or isolated human islet beta β-cells [6], [18]. These results suggest that GABA, via activation of GABA_A_R, induces membrane potential depolarization in pancreatic INS-1 cells.

We then performed intracellular Ca^2+^ measurements to determine if GABA-induced membrane depolarization increases intracellular Ca^2+^ levels in INS-1 cells. As shown in **Figure 1C**, GABA (30 µM) evoked a steep rise in intracellular Ca^2+^ concentrations, which declines and then persists at a stable level during the course of the 20 to 30 min recording period. These results indicate that GABA induces membrane depolarization that is associated with increased intracellular Ca^2+^ in a population of pancreatic β-cells.

### Insulin inhibits I_GABA_ in INS-1 cells

**Figure 2:**
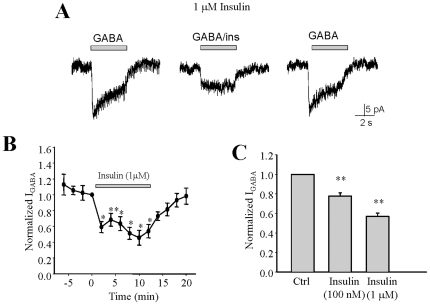
GABA-evoked currents (I_GABA_) is inhibited by insulin in INS-1 cells. GABA-evoked inward current was measured by means of a computer-controlled multi-barrelled perfusion system, in two-minute intervals, under voltage-clamp conditions. Representative traces of GABA-evoked currents in the absence and presence of insulin (100 nM, (A), 1 µM, (B)) in the same INS-1 cell. A' and B' represents the average of I_GABA_ from separated experiments. (C) Normalized average I_GABA_ during the course of experiment (control = average of first 4 I_GABA_, insulin = average of I_GABA_ in the presence insulin at indicated concentrations). Data were mean ± SE. *p<0.05 ** p<0.01, n = 6.

Under voltage-clamp mode, perfusion of GABA evoked typical bicuculline-sensitive GABA current (**Figure 2A**). We next determined the effect of insulin on GABA-induced current (I_GABA_). Treatment of INS-1 cells with insulin (100 nM) significantly decreased I_GABA_ by 22% (P<0.05, n = 8). The insulin-induced suppression of I_GABA_ was more prominent when the insulin concentration was increased, for instance a reduction of 43% in I_GABA_ was achieved with 1 µM insulin (**Figure 2A, 2B** p<0.05, n = 5). Thus, insulin-induced suppression of I_GABA_ was dose-dependent (**Figure 2C**).

### Zinc-free insulin inhibits I_GABA_ in INS-1 cells

**Figure 3:**
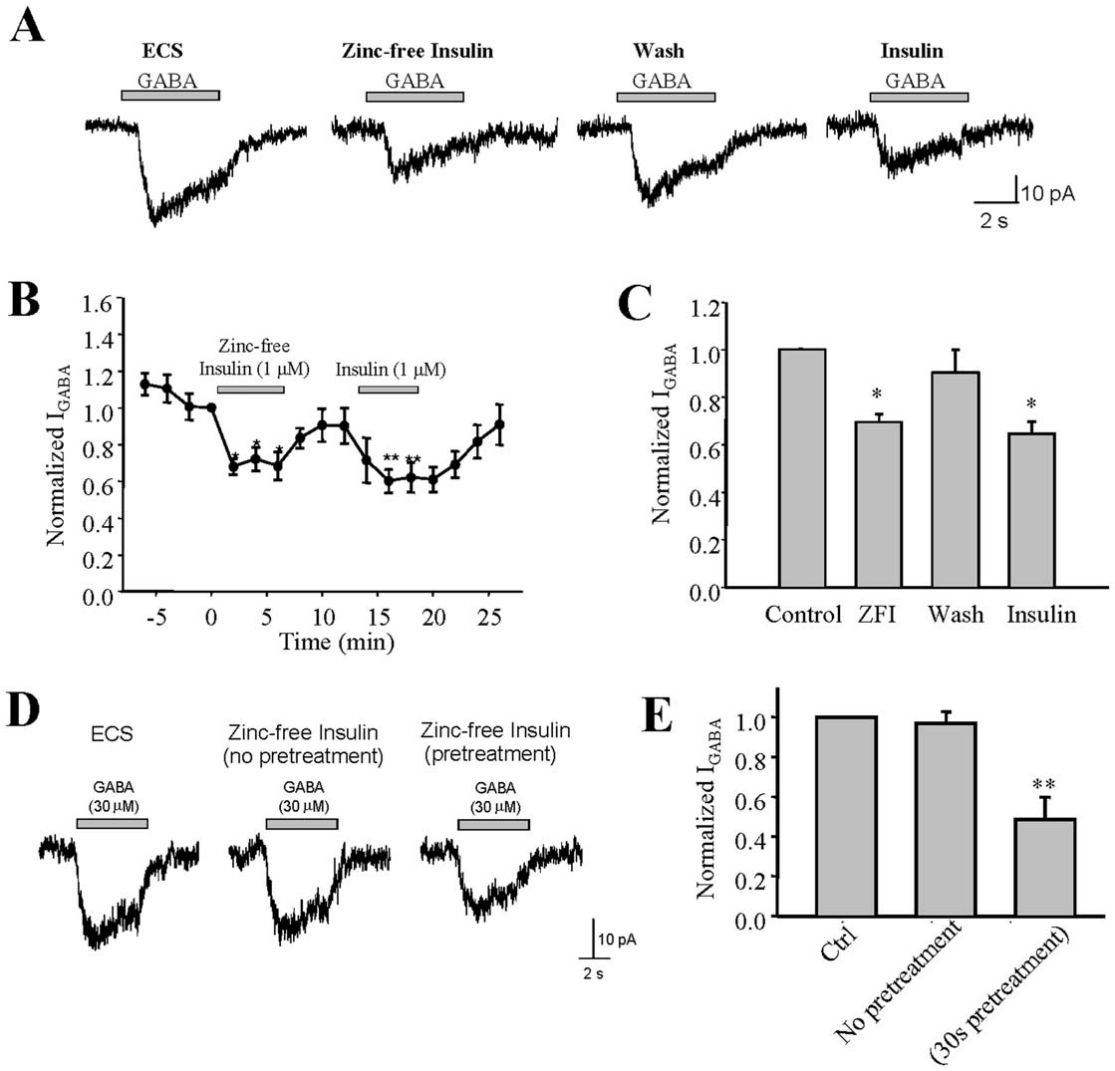
Zinc-free insulin inhibits GABA-evoked currents in INS-1 cells. (A) Representative traces of GABA-evoked currents in the absence and presence of zinc-free insulin and regular insulin in the same INS-1 cell. (B) The average of I_GABA_ from separated experiments. (C) Normalized average I_GABA_ was separately recorded during the course of experiment (control = average of first 4 I_GABA_, ZFI = average of I_GABA_ in the presence zinc-free insulin, insulin = average of I_GABA_ in the presence of insulin after washing out). (D) Representative traces of I_GABA_ obtained from when GABA was applied simultaneously with insulin or 30 seconds after insulin pre-treatment. (E) Normalized average I_GABA_ of separated experiments as described in (C). Data were mean ± SE. *p<0.05, n = 5.

Clinically-used insulin contains zinc [21] and zinc inhibits I_GABA_ in neurons [22] by directly binding to GABA_A_R channel and lowering its open probability [23]. Therefore, we used zinc-free insulin to verify the suppressive effects of insulin on I_GABA._ Our result showed that the zinc-free insulin also suppressed I_GABA_ in INS-1 cells (**Figure 3A**, p<0.05). The efficacy of zinc-free insulin was similar to that of regular insulin (**Figures 3B and 3C**). However, the suppression of I_GABA_ by insulin disappeared when zinc-free insulin was applied simultaneously with GABA (**Figures 3D and 3E**, p>0.05, n = 3). We found that the reduction of I_GABA_ was seen only when zinc-free insulin was pre-applied to the cell (i.e., >30 seconds) prior to GABA application (**Figures 3D and 3E**, p<0.05, n = 3). These results suggest that zinc-free insulin-induced inhibition of I_GABA_ is not a result of direct blockade of GABA_A_R channels, but rather through a signaling process.

### Insulin-induced inhibition on I_GABA_ is PI3-K/Akt independent

**Figure 4:**
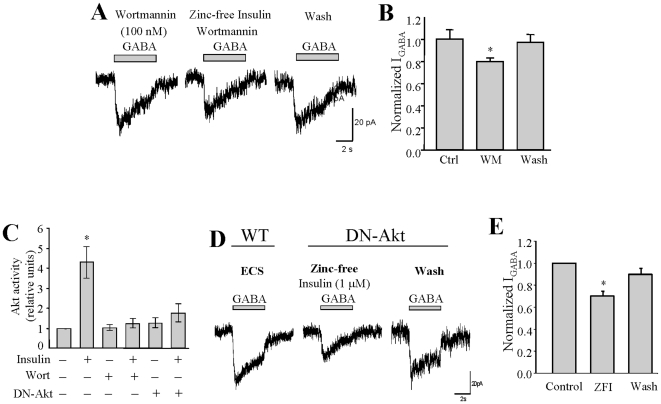
Insulin-induced inhibition of I_GABA_ in INS-1 cells is PI3-K/Akt independent. (A) Representative traces of GABA-evoked currents in the absence and presence of zinc-free insulin (1 µM) along with PI3-K inhibitor wortmannin (100 nM). (B) Normalized average of I_GABA_ from separated experiments. (C) Akt activity determined by Western Blotting using anti-phospho Akt (S473) in cells treated without or with wortmannin (Wort), or in the cells transfected with dominant-negative Akt (DN-Akt). (D) Representative traces of GABA-evoked currents in cells expressing DN-Akt in the absence and presence of zinc-free insulin (ZFI,1 µM). (E) Average I_GABA_ from separated time-course experiments. Data were mean ± SE. *p<0.05, ** p<0.01, n = 5.

PI3-K is a key signaling molecule that mediates the trophic effects of insulin [24]. We therefore examined whether insulin-induced inhibition of I_GABA_ requires involvement of PI3-K. INS-1 cells were pretreated with 100 nM of the specific PI3-K inhibitor wortmannin for 10 minutes, and then treated with 1 µM zinc-free insulin prior to measurement of I_GABA_. As shown (**Figures 4A and 4B**), the zinc-free insulin-induced inhibition of I_GABA_ persisted in the presence of PI3-K inhibitor_,_ which is suggestive of a PI3-K independent process. To confirm this finding, we transfected INS-1 cells with a vector expressing a dominant-negative form of Akt (DN-Akt) and tagged with green fluorescent protein (GFP) [12]. The dominant negative effect of DN-Akt was determined in parallel experiments by Western Blot using anti-phospho-Akt (Ser473) antibody in either transfected or non-transfected INS-cells treated with or without inhibitors as indicated (**Figure 4C**). Application of zinc-free insulin (1 µM) to the transfected INS-1 cells still caused a remarkable reduction of I_GABA_ (**Figures 4D**). Normalized I_GABA_ from separated experiments showed zinc-free insulin reduced I_GABA_ by approximately 30% in INS-1 cells expressing DN-Akt (**Figure 4E**, n = 5, p<0.05). These observations suggest that zinc-free insulin-induced inhibition on I_GABA_ is not sensitive to the PI3-K/Akt inhibition.

### Insulin does not alter the localization of GABA_A_R at the INS-1 plasma membrane

**Figure 5:**
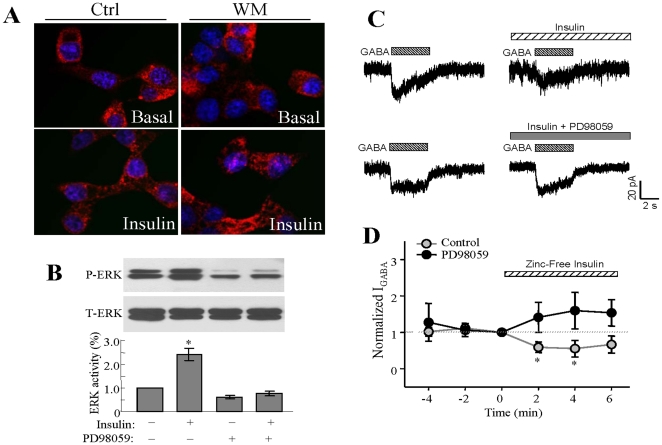
Insulin suppresses I_GABA_ which is not associated with GABA_A_R membrane relocalization and is ERK-dependent. (A) Confocal microscopic image of INS-1 cells immunostained for GABA_A_Rs using anti-GABA_A_R β_2/3_ mouse IgG and Cy3-conjugated secondary antibody (red) with DAPI-nuclear staining (blue). Cells were treated with or without insulin, in the presence or absence of PI3-K inhibitor wortmannin. (B) Insulin (100 nM, 5 min) stimulated ERK phosphorylation in INS-1 cells, which was blocked by pre-treatment of the cells with PD98059 (20 µM, 10 min). (C) Representative traces of GABA-evoked currents in the absence and presence of zinc-free insulin (0.6 µM) with or without PD98059 (20 µM). (D) Normalized average of I_GABA_ from separated experiments. Data were mean ± SE. *p<0.05, ** p<0.01, n = 5–6.

We previously demonstrated that insulin enhances the insertion of GABA_A_R into the plasma membrane in neuron [25] and α-cell [12]. We thus investigated whether insulin could alter GABA_A_R membrane expression in INS-1 cells by immunostaining using antibody against the GABA_A_Rβ_2/3_ subunits. As shown, insulin (1 µM, 15 min) did not alter the staining profile of GABA_A_Rβ_2/3_ subunits (**Figure 5A**) in INS-1 cells treated with or without wortmannin, suggesting that insulin-induced suppression of I_GABA_ is not related to GABA_A_R redistribution in INS-1 cells.

### Insulin-induced inhibition of I_GABA_ is ERK dependent

Activation of the MRK/ERK pathway represents another important branch of the insulin receptor signal transduction pathway in pancreatic β-cells [26]. We performed experiments to examine whether or not insulin-induced inhibition of I_GABA_ requires activation of MEK/ERK. We found that incubation of INS-1 cells with insulin (100 nM, 5 min) resulted in rapid ERK1/2 phosphorylation, which was blocked by the MEK/ERK inhibitor PD98059 (20 µM) (**Figure 5B**). Perforated patch clamp recordings were then performed in the control INS-1 cells and INS-1 cells that were pretreated with PD98059 (20 µM, 10 min). We found that application of zinc-free insulin to the cells failed to inhibit I_GABA_ in the presence of PD98059 (**Figures 5C, 5D** **;** p<0.05, n = 6). This result suggests that insulin suppresses GABA_A_R function via activation of the MEK/ERK pathway.

### Insulin inhibits GABA-induced INS-1 cell secretion

**Figure 6:**
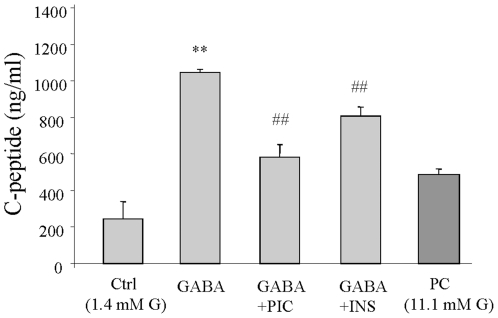
GABA enhances insulin secretion which is attenuated by insulin in INS-1 cells. Insulin secretion was evaluated by C-peptide RIA. Cells were serum-starved in KRB buffer containing 1.4 mM glucose for 60 min prior to the RIA. The RIA was conducted using cells which had their culture medium replaced with fresh KRB buffer containing 1.4 mM glucose (or 11.1 mM glucose as positive control, PC), in the presence of GABA (30 µM), with or without either picrotoxin (Pic, 50 µM) or insulin (Ins, 1 µM) for 120 min. Data were mean ± SE, from three independent experiments with each sample counted in triplication. ** p<0.01 (Ctrl vs GABA), ## p<0.01 (GABA vs GABA+Bic or GABA+Ins).

We next conducted C-peptide radioimmunoassays (RIA) to determine whether or not the effect of insulin on the modulation of GABA-GABA_A_R system has an impact on GABA_A_R-mediated secretory function in INS-1 cells. As shown, consistent with our previous findings [15], GABA (30 µM) significantly increased C-peptide secretion in INS-1 cells, which was diminished by the GABA_A_R antagonist picrotoxin (50 µM) (**Figure 6**, p<0.01, n = 3), suggesting that GABA-induced insulin secretion in the β-cells is mediated by GABA_A_R. Furthermore, pre-treatment of the INS-1 cells with insulin (1 µM) resulted in a statistically-significant decrease in GABA-induced C-peptide secretion (**Figure 6** **,** p<0.01, n = 3). These results suggest that insulin-mediated inhibition of I_GABA_ is related to down-regulation in GABA-induced insulin secretion in INS-1 cells.

## Discussion

Pancreatic β-cells produce a large amount of GABA [27], whereas GABA_A_Rs are expressed in both β-cells [6], [15] and α-cells [7], [12]. In α-cells, GABA hyperpolarizes the membrane potential and suppresses glucagon secretion [7], [12], via a mechanism involving PI3-K/Akt signaling dependent GABA_A_R plasma membrane translocation [12]. In contrast, we and others demonstrated that GABA depolarizes β-cells and stimulates insulin secretion from these cells [6], [15]. These observations suggest that GABA, as a paracrine or autocrine factor plays an important role within pancreatic islets in the regulation of islet cell secretion and function. In the present study, we sought to investigate how insulin affects GABA-GABA_A_R system in the β-cells and thereby modulates its secretory pathways.

In INS-1 cells, glucose induces a gradual and sustained depolarization, whereas GABA produces rapid and bicuculline- or picrotoxin-sensitive membrane depolarization, associated with remarkable increases in intracellular Ca^2+^ concentration and insulin secretion. A recent study by Braun *et al.* suggested that glucose stimulates feed-forward release of GABA from the β-cells [6]. Furthermore, the GABA-stimulated insulin release appears to be glucose concentration-dependent [6], [15]. Of note, our results showed that GABA_A_R antagonist picrotoxin attenuated about 50% of the GABA-induced C-peptide release. This is likely due to the fact that GABA-stimulated insulin secretion in the β-cells is partially contributed by activation of B-type GABA receptor (GABA_B_R) [28]. These observations suggest that the autocrine GABA-GABA_A_R system in β-cells constitutes an effective signaling component of the glucose-sensing machinery.

The opposite effects of GABA in the two types of islet endocrine cells are likely because β-cells and α-cells have different Cl^-^ reversal potential (*E_Cl_*). The direction of Cl^-^ flow upon opening of the GABA_A_R channel is dependent on the electrochemical driving force which is determined by the resting membrane potential and the *E_Cl_* [29]. For example, in the early developing brain, GABA induces depolarizing effects in immature neurons [30], while it exerts inhibitory effects by hyperpolarizing the membrane potential in mature neurons of the adult brain [31]. The switch from excitation to inhibition of GABA_A_R activation is due to a shift of *E_Cl_* which is controlled by increased activity of K^+^-Cl^-^ co-transporter-2 (KCC2) in the brain during development [32]. In this regard, functional KCC has been identified in pancreatic α-cells, but not in the β-cells [33], [34].

Regular human insulin is a complex of insulin and zinc [35]. The finding that zinc-free insulin suppressed I_GABA_ to a degree similar to that of regular insulin suggests that the inhibitory effects of insulin on I_GABA_ is dependent on the insulin peptide. It is interesting to note that application of zinc-free insulin together with GABA did not inhibit I_GABA_, whereas pre-treating the cell for at least 30 seconds with zinc-free insulin inhibited I_GABA_. These results suggest that insulin-induced inhibition of I_GABA_ in INS-1 cells requires insulin signaling processes. Such inhibitory effects of insulin on GABA-induced current was also observed in the non-islet β-cells [36] The potentiating effect of insulin on I_GABA_ in neurons and α-cells is attributed to GABA_A_R insertion into the plasma membrane, which occurred about 10-15 min after insulin treatment [12], [25]. Under similar experimental conditions, however, we did not observe increased GABA_A_R localization at the plasma membrane upon insulin treatment in INS-1 cells. Furthermore, unlike in the α-cells, where the insulin-enhanced I_GABA_ is PI3-K/Akt dependent, our data does not suggest the involvement of PI3-K/Akt signaling in the inhibition of I_GABA_ by insulin in the β-cells. In contrast, MEK/ERK inhibitor PD98059 blocked the inhibitory effect of insulin on GABA-induced current, suggesting that insulin regulates GABA_A_R function in INS-1 cells via activation of the MEK/ERK signaling pathway.

The explanation for the opposite effects of insulin on GABA_A_R in α- and β-cells is largely unknown, although it may be due to the different subunit composition of GABA_A_R in the two types of islet cells [12], [15]. It is interesting to note that in neurons, activation of the insulin-PI3-K signaling pathway enhances I_GABA_ due to the increase in cell surface-localized GABA_A_R, whereas activation of insulin receptor with ERK kinase causes inhibition of I_GABA_ through phosphorylation of a specific subunit of GABA_A_R [36], [37]. Particularly, α-subunits of the GABA_A_R have a putative phosphorylation site for ERK [37]. Presumably, such phosphorylation occurs on an intracellular site allowing immediate allosteric modifications of GABA_A_R.

Given the relatively rapid inhibitory effect of insulin on I_GABA_, it is possible that insulin may also act as a non-competitive inhibitor of the GABA_A_R in the β-cells, as has been observed in non-β-cells [36]. In relation to this notion, it has been reported that a direct receptor-receptor interaction occurs between GABA_A_R and dopamine D5 receptor, which affects the GABA_A_R activation [38]. Further study is warranted to test if there is an interaction between insulin receptor and GABA_A_R, and to determine the molecular mechanism by which insulin modulates GABA-GABA_A_R signaling in the β-cells.

In INS-1 cells, insulin suppresses I_GABA_ and decreases GABA-mediated insulin secretion in the β-cells which suggests that insulin may utilize the GABA-GABA_A_R system to constitute a feedback mechanism for the β-cell secretion. Our findings are in a good agreement with previous observations suggesting that activation of insulin receptor inhibits insulin secretion in the β-cells [39]. Conversely, inhibition of PI3-K signaling pathways enhances insulin secretion in the β-cells [40], [41]. A study by Khan et al suggested that insulin inhibits insulin secretion through activation of K_ATP_ channels in the β-cells [42]. A study by Jimenez-Feltstrom and colleagues [43] suggested that the effect of insulin on I_GABA_ is insulin-dose dependent, exemplified by the observation that, insulin, at low concentrations (i.e., from 0.05 to 0.1 nM) stimulated insulin release, while at concentrations higher than 250 nM, insulin inhibited insulin secretion from the β-cells.

It should be noted that under certain circumstance, effects of insulin on I_GABA_ are excitatory [16]. These previous reports that describe the stimulatory effects of insulin on β-cell secretory process were mostly supported by experiments involving β-cells from organisms with genetic knockout or overexpression of the insulin receptor [44]–[48], The different outcomes imply that the modulation of insulin on the GABA-GABA_A_R system in the β-cells may be dependent on their metabolic status.

The physiological relevance of GABA signaling in the regulation of islet β-cell function has yet to be fully identified. We demonstrated recently that the depolarizing effects of GABA may lead to activation of PI3-K/Akt dependent cell growth and survival pathways in the β-cells [18]. Insulin is an important positive autocrine regulator of β-cell growth and survival [17], [49]. GABA, when co-released with insulin [6], synergistically enhances insulin-stimulated cell growth and survival pathways in the β-cells [18]. In support of previous findings that insulin is a negative regulator of insulin secretion [39]–[42], [50], our data suggest that insulin utilizes the autocrine GABA-GABA_A_R pathway to operate its negative feedback suppression in the β-cells.

Such a negative feedback modulator appears to be important for maintaining islet hormones at appropriate levels [51]. It is conceivable that basal insulin may serve as a maintenance signal that primes the β-cell to respond to subsequent glucose stimulus, insulin may utilize GABA-GABA_A_R system to inhibit further release at the peak of the exocytotic event, particularly, at very high local insulin concentration. Previous euglycemic hyperinsulinemic clamp studies in humans suggest that this negative short-loop insulin-β-cell feedback is an important mechanism in maintaining appropriate β-cell secretion, since inadequate feedback suppression is found in obese patients, and may partly account for their prevailing hyperinsulinemia [52]. Given that autocrine insulin action is critical in maintaining normal β-cell function [16], [17], and that β-cell insulin resistance can deteriorate β-cell function that accelerates the progression of diabetes [53], [54], future studies are required to determine whether the impairment of the autocrine insulin-GABA-GABA_A_R signaling contributes to β-cell insulin resistance in type 2 diabetes.
